# The Effect of Decomposed PbI_2_ on Microscopic Mechanisms of Scattering in CH_3_NH_3_PbI_3_ Films

**DOI:** 10.1186/s11671-019-3022-y

**Published:** 2019-06-18

**Authors:** Dan Shan, Guoqing Tong, Yunqing Cao, Mingjun Tang, Jun Xu, Linwei Yu, Kunji Chen

**Affiliations:** 1 National Laboratory of Solid State Microstructures and School of Electronic Science and Engineering and Collaborative Innovation Center of Advanced Microstructures, Nanjing University, Nanjing, 210093 China; 2 School of Electronic and Information Engineering, Yangzhou Polytechnic Institute, Jiangsu, 225127 China; 3 Huafu Energy Storage New Technique Co., Ltd., Jiangsu, 225600 China; 4 Energy Materials and Surface Sciences Unit, Okinawa Institute of Science and Technology Graduate University, Okinawa, Japan; 5 College of Physical Science and Technology, Yangzhou University, Jiangsu, 225009 China

**Keywords:** MAPbI_3_ film, Hall measurement, Scattering mechanism, Grain boundary

## Abstract

Hybrid organic-inorganic perovskites (HOIPs) exhibit long electronic carrier diffusion length, high optical absorption coefficient, and impressive photovoltaic device performance. At the core of any optoelectronic device lie the charge transport properties, especially the microscopic mechanism of scattering, which must efficiently affect the device function. In this work, CH_3_NH_3_PbI_3_ (MAPbI_3_) films were fabricated by a vapor solution reaction method. Temperature-dependent Hall measurements were introduced to investigate the scattering mechanism in MAPbI_3_ films. Two kinds of temperature-mobility behaviors were identified in different thermal treatment MAPbI_3_ films, indicating different scattering mechanisms during the charge transport process in films. We found that the scattering mechanisms in MAPbI_3_ films were mainly influenced by the decomposed PbI_2_ components, which could be easily generated at the perovskite grain boundaries (GBs) by releasing the organic species after annealing at a proper temperature. The passivation effects of PbI_2_ in MAPbI_3_ films were investigated and further discussed with emphasis on the scattering mechanism in the charge transport process.

## Background

Hybrid organic-inorganic perovskite (HOIP) materials have recently emerged as highly efficient optoelectronic materials and are being intensively investigated and developed for photovoltaics, photo-detections, light-emitting diodes, and laser devices [1–6]. The perovskite solar cells have gradually emerged in the center of photovoltaic filed because of their power conversion efficiency achieving over 20% during the past 8 years, as well as their cost-effective and scalable processability [7–14]. The investigations on HOIP materials (e.g., CH_3_NH_3_PbX_3_, X = Cl, Br, I) have revealed their great potentials for photovoltaic applications due to optimum band gap, high absorption coefficient, high carrier mobility, and diffusion length on the order of hundreds of nanometers to micrometers in films [15–19]. At the core of any optoelectronic devices lie the electronic properties, especially the scattering mechanism governing charge transport process. There have been many works allowing us to understand HOIP charge transport characteristics. It is apparent that the carrier mobilities of HOIP materials, which are only within the scope of 1~10 cm^2^/V s [20–22], are usually limited by the scattering mechanism. The *T*^−1.3^ to *T*^−1.6^ dependence of the mobilities on temperature have been observed by several groups, which are close to the *T*^−1.5^ dependence usually assumed for the scattering of acoustic phonon [23, 24]. Furthermore, the scattering from grain boundaries (GBs) on charge transport in HOIPs remains unclear. The impacts of GBs with different studies usually reach conflicting conclusions. Edri et al. found a barrier in potential across the GBs in the dark, which could be reduced during the illumination [25]. Yun et al. also revealed the generation of a very small photo-voltage at GBs, but the reduced photoluminescence efficiency was found due to a deep trapping at GBs [26]. From the above introduction, we can know that although HOIP device efficiencies have increased rapidly, an understanding of the charge transport mechanisms of these materials and their underlying physical mechanisms is only starting to carry out.

In this work, the vapor solution reaction method was employed to construct compact and uniform CH_3_NH_3_PbI_3_ (MAPbI_3_) films. The microscopic mechanism of scattering during the charge transport process in MAPbI_3_ films was evaluated via temperature-dependent Hall measurements. Two different behaviors of temperature-dependent Hall mobilities could be identified in the MAPbI_3_ films before and after thermal annealing. It is confirmed that the decomposed PbI_2_ located at the GBs, which is usually converted from MAPbI_3_ upon thermal annealing at a proper temperature, plays an important role in the charge transport process in MAPbI_3_ films. The different scattering mechanisms combining the microstructure of MAPbI_3_ films were discussed, and the possible physical mechanisms were further proposed.

## Methods

MAPbI_3_ films were fabricated by the vapor solution reaction method as our previous works [27, 28]. The PbI_2_ powder (purchased from Xi’an Polymer Light Technology Corp, 99.99%.) was first dissolved in the *N*,*N*-dimethylformamide (DMF, Aladdin, 99.9%) solvent with a concentration of 1 mol/mL and stirred at 70 °C for 3 h. Then, the PbI_2_ film was coated on the substrates by spin-coating with a speed of 4000 rpm, 30 s, and annealed at 70 °C for 10 min. The PbI_2_ films and MAI powder were separately placed in two different zones of the tubular furnace equipment with a vacuum system. After pumping for 10 min, the MAI power and PbI_2_ films are heated to 180 °C and 140 °C, respectively, and kept these temperatures for more than 100 min. Finally, the pre-prepared MAPbI_3_ films with darkened color were annealed at different temperatures (100 °C, 120 °C, and 145 °C) for 1 h, after being washed with isopropanol. All the procedures were carried out in the open air with a humidity of ~ 45%.

The microstructures of MAPbI_3_ films were measured by using X-ray diffraction (XRD) (model: MXP-III, Bruker Inc.) and scanning electron microscopy (SEM) (model: S-3400 N-II, Hitachi Inc.). The fluorescence decay curves from time-resolved photoluminescence (TRPL) measurements were recorded by a fluorescence spectrophotometer based on the time-correlated single photon counting (model: FLS920, Edinburgh Inc.). Temperature-dependent Hall measurements (model: LakeShore 8400 series, LakeShore Inc.) were performed with coplanar configurations by using Al electrodes prepared by thermal evaporation technique. Hall mobilities could be obtained from Hall effect measurements, carried out in a standard van der Pauw configuration by using an electromagnet with a magnetic induction of 0.6 T. All the temperature dependence measurements were taken during heating in the temperature range from 300 to 350 K with a step of 10 K in argon ambient.

## Results and Discussion

**Fig. 1:**
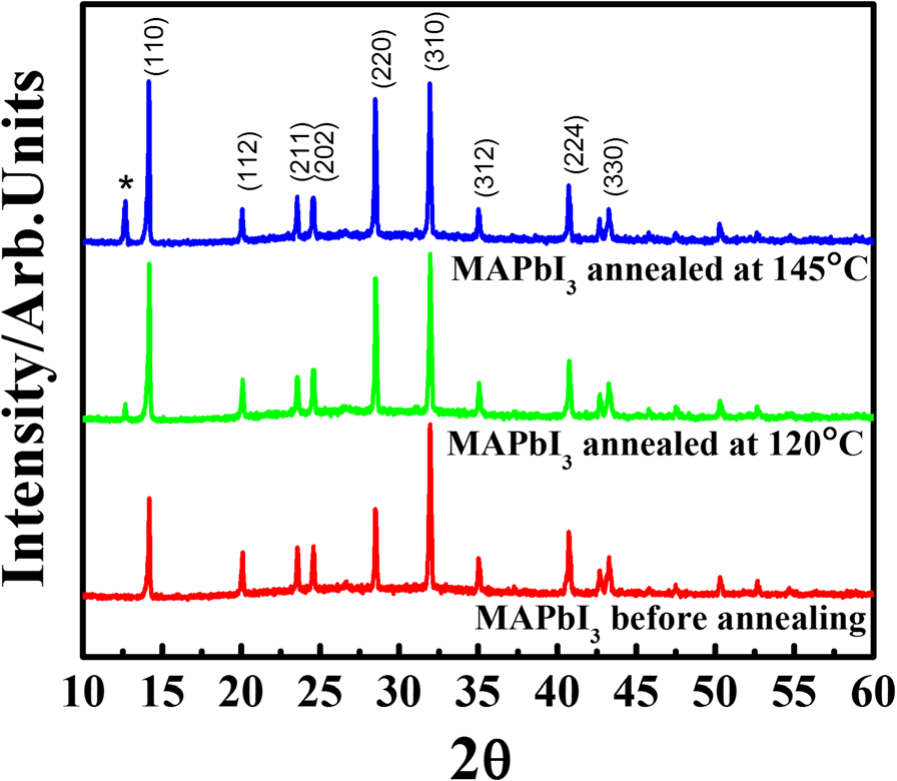
The XRD pattern for MAPbI_3_ films before and after annealing at 120 °C and 145 °C

The morphology evolution of MAPbI_3_ films was firstly investigated by XRD measurement. The XRD patterns for the MAPbI_3_ films before and after annealing are shown in Fig. 1. It can be clearly seen that the samples before annealing and after annealing at 120 °C are composed of pure perovskite phase, which reveals the MAPbI_3_ characteristic peaks at 14.04°, 28.42°, and 43.08° corresponding to the (110), (220), and (330) planes of MAPbI_3_, respectively [29]. However, it is found that the sample after annealing at 145 °C is not pure MAPbI_3_ film. A new characteristic diffraction peak at 12.56° appears in the corresponding film, which could be observed by the (001) planes of PbI_2_. There have been a lot of previous reports suggesting that a structural conversion from MAPbI_3_ to PbI_2_ occurs mostly in MAPbI_3_ films upon thermal annealing [30–32]. According to these reports, we believe that MAPbI_3_ films could be decomposed via heating above 145 °C in this work, where CH_3_NH_3_I species escape from MAPbI_3_ film to form the PbI_2_ phase. This indicates the weakly bonded nature between the organic and inorganic species in MAPbI_3_ films [33].

**Fig. 2:**
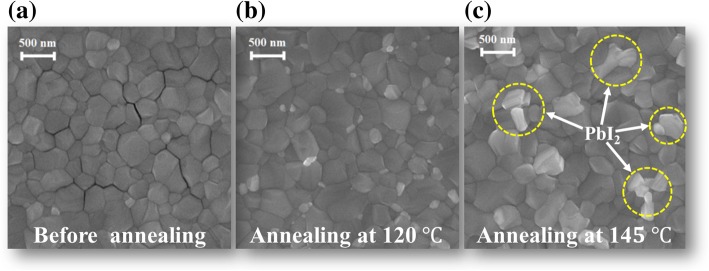
The SEM images for MAPbI_3_ films before (**a**) and after annealing at 120 °C (**b**) and 145 °C (**c**)

The SEM images further gave a deep insight into the morphology evolution of MAPbI_3_ films. In Fig. 2a–c, all the films present a compact and conformal structure. However, an amount of newly formed species occurring at GBs is emerged in the MAPbI_3_ film annealed at 145 °C, which shows relatively bright contrast compared to the adjacent grains. These newly formed species have been reported previously in similar works where they were speculated to be PbI_2_ components, which is consistent with the conservation of PbI_2_ signals in the corresponding XRD pattern as we discussed before [33]. From these findings, we can conclude that a compositional change could occur in the MAPbI_3_ film annealed above 145 °C. By releasing the organic species during annealing, PbI_2_ components are decomposed and parts of them are located at the perovskite GBs according to both XRD and SEM results.

**Fig. 3:**
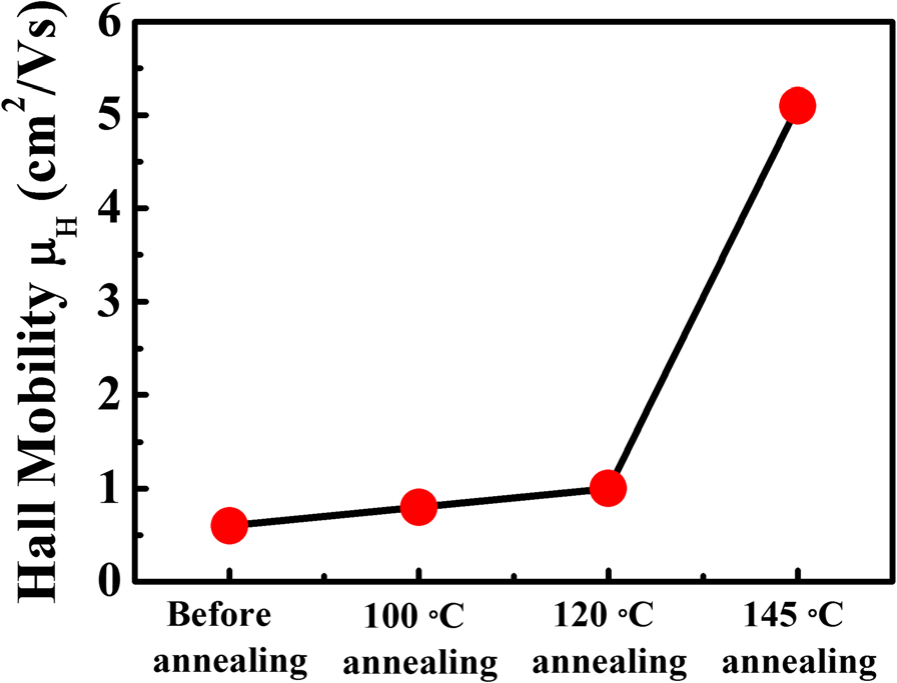
Hall mobilities of all the MAPbI_3_ films at room temperature

An understanding of the charge transport properties in MAPbI_3_ films is highly important as the mobility usually dominates device performance. In this work, Hall mobilities of all the MAPbI_3_ films were measured as shown in Fig. 3. At room temperature, Hall mobilities are around 0.6~1 cm^2^/V s for the unannealed, 100 °C- and 120 °C-annealed MAPbI_3_ films, which are consistent with the previous reports [20, 34]. However, increased Hall mobility reaching to 5 cm^2^/V s is found in the 145 °C-annealed MAPbI_3_ film, which is nearly one order of magnitude higher than that of the unannealed one. As we know, mobility is usually influenced by the dominant scattering mechanism governing the charge transport process. Such increased Hall mobility probably reflects a reduction of scattering during the charge transport process in the 145 °C-annealed MAPbI_3_ film. Yang et al. once investigated the surfaces and GBs in MAPbI_3_ films via scanning Kelvin probe microscopy (SKPM), which is used to determine the surface potential difference between GBs and inner grains in films. It was found that the MAPbI_3_ film without thermal annealing exhibited a higher surface potential at the GBs than that at the bulk, which was usually reported in the previous works [35–37]. In contrast, the surface potential at the GBs was obviously reduced after annealing at 150 °C. They considered that the decreasing of surface potential resulted from the passivation effect of newly formed PbI_2_ phases, which could suppress the barrier of GBs to some extent thus reduced the scattering from GBs [33, 38]. Therefore, with the decomposed PbI_2_ occurring after annealing at 145 °C in this work, the increased Hall mobility can be attributed to the passivation effect of the decomposed PbI_2_ at GBs. As the Hall measurements characterize the charge transport property of the entire film, it is inferred that the decomposed PbI_2_ passivates the GBs and reduces the energy barrier between grain domains, facilitating the charge transportation in the plane direction [39].

**Fig. 4:**
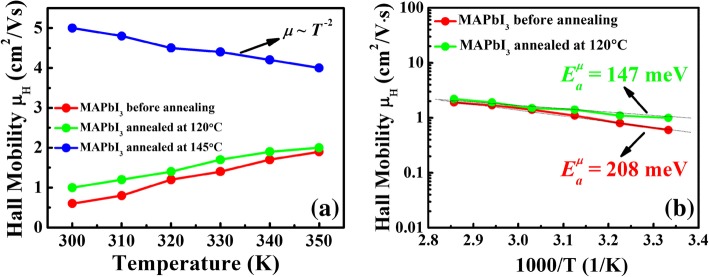
**a**, **b** Temperature-dependent Hall mobilities of the MAPbI_3_ films before and after annealing at 120 °C and 145 °C

In order to further study the passivation effect of decomposed PbI_2_ locating at GBs in MAPbI_3_ films, temperature-dependent Hall mobilities were introduced to investigate the scattering mechanism in the MAPbI_3_ films before and after annealing. Hall mobilities-temperature behaviors within the temperature range from 300 to 350 K are shown in Fig. 4a. It is clearly seen that the mobility is increased with temperature for the un- and 120 °C-annealed MAPbI_3_ films. As we know, the GBs in the films with grain sizes on the micrometer scale play an important role in the charge transport process and the carrier mobility is limited by the potential energy barriers at GBs [40]. Such GBs with a large number of defects can trap the carriers and form the electrically charged states, which impede the motion of carriers from one crystallite to another and thus reduce the mobility [41]. With increasing the temperature, the carriers gain the kinetic energy to overcome the potential barriers and the carrier mobility can be increased accordingly [42]. Consequently, it is indicated that a GB scattering governs the charge transport process [43]. Seto et al. established the theoretical model to describe the GB scattering process and Hall mobility *μ*_0_ shows the thermally activated behavior as below:

$$ {\mu}_H(T)={\mu}_0\exp \left(-{E}_B/{k}_BT\ \right) $$

where *k*_*B*_ is the Boltzmann’s constant, *μ*_0_ is the exponential prefactor, and *E*_*B*_ is the activation energy which corresponds to the potential energy barrier height [44]. The relationship between *ln μ*_*H*_ and 1000/*T* is given within the temperature from 300 to 350 K as shown in Fig. 4b while the potential barrier height *E*_*B*_ of GBs can be deduced from the slope of the linear fitting. It can be found that the potential barrier height *E*_*B*_ of GBs is about 208 meV for the unannealed MAPbI_3_ film and slightly reduces to 147 meV after annealing at 120 °C, which is almost in accordance with the previous reports [45]. However, after annealing at 145 °C, the MAPbI_3_ film where the decomposed PbI_2_ locating at the GBs exhibits a different temperature-dependent behavior. It is interesting to find that the mobility is decreased with the temperature increasing, which finally exhibits a *T*^−2.0^ dependence. Such close to *T*^−1.5^ dependence is usually assumed for the acoustic phonon scattering [23, 24]. It thus appears that the charge transport process in the 145 °C-annealed MAPbI_3_ film is no longer dominated by the GB scattering, of which the acoustic phonon scattering would be instead in the charge transport process. Therefore, we could convince that the decomposed PbI_2_ locating at the GBs acts as a passivation layer between the grains and suppresses the potential barrier of GBs, thus leading to the change of scattering mechanism in the charge transport process from GB scattering to acoustic phonon scattering.

**Fig. 5:**
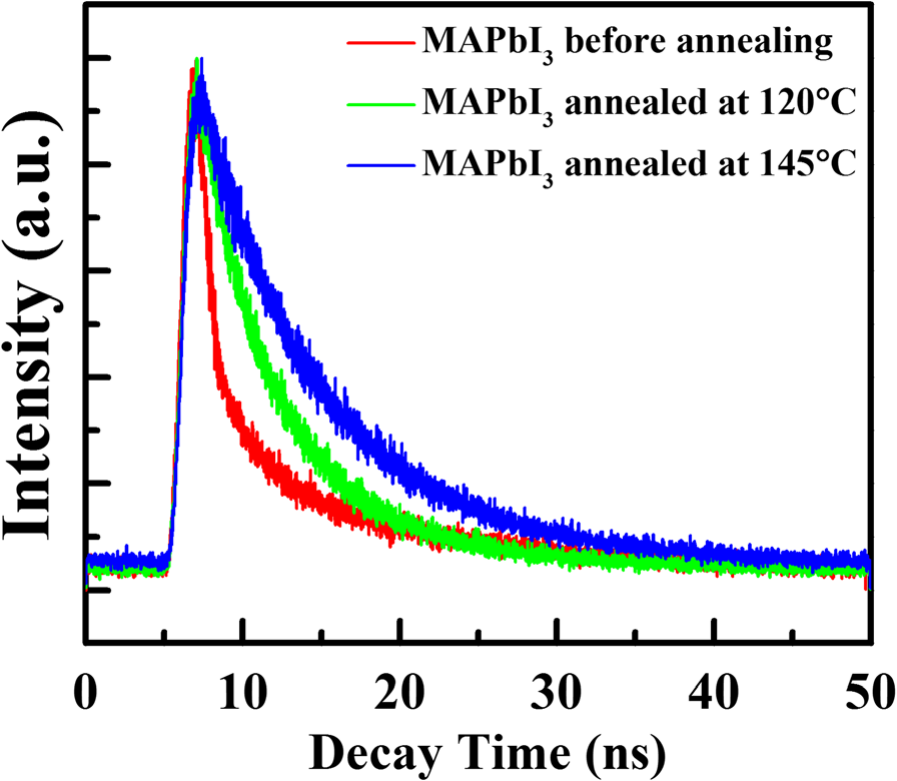
The TRPL decay spectra of the MAPbI_3_ films before and after annealing at 120 °C and 145 °C

Furthermore, the TRPL decay was employed and performed on the MAPbI_3_ films before and after thermal annealing, and the emission lifetime could be obtained by fitting the fluorescence emission decay spectra using the exponential function. The corresponding fluorescence emission lifetime would reflect the charge recombination behavior in the corresponding MAPbI_3_ films. Figure 5 shows the TRPL decay spectra, and Table 1 displays the corresponding lifetime of MAPbI3 films. The fluorescence emission decay curves are fitted with two-component exponential decay which exhibits the same scale of lifetime to the reported PL decay in MAPbI_3_ films [46]. The fast decay component, *τ*_1_, might come from the surface or interface charge recombination lifetime, and the long decay component, *τ*_2_, could be attributed to the bulk charge recombination lifetime [47, 48]. It is found that the long decay component *τ*_2_ shows little variation in the MAPbI_3_ films before and after thermal annealing. However, the fast decay component *τ*_1_ is increased from 1.39 ns for unannealed sample to 6.05 ns for 145 °C-annealed one, proving a reduction of surface or interface recombination, which finally results in an increase of reduced emission lifetime *τ* after increasing thermal annealing temperature. In the previous works, Wang et al. also investigated the charge recombination in MAPbI_3_ films by analyzing the emission lifetime. They found that longer emission lifetime would indicate the enhanced suppression of the charge recombination, which could be attributed to the decomposed PbI_2_ effectively passivating the charge traps at GBs in MAPbI_3_ films [40]. Therefore, in this work, the enhanced *τ* could be ascribed to the increasing passivation effect of the decomposed PbI_2_ locating at GBs which fills the GBs and suppresses the interface charge recombination in MAPbI_3_ films. This is another powerful evidence for the passivation effect of the decomposed PbI_2_ at the MAPbI_3_ GBs.

**Table 1:** The emission lifetime of MAPbI_3_ films before and after annealing at 120 °C and 145 °C

TRPL	*τ*_1_ (ns)	*τ*_2_ (ns)	*τ* (ns)
MAPbI_3_ before annealing	1.39	11.38	7.48
MAPbI_3_ annealed at 120 °C	3.02	10.48	7.72
MAPbI_3_ annealed at 145 °C	6.05	10.42	8.53

## Conclusions

In summary, MAPbI_3_ films were fabricated by a vapor solution reaction method. The microstructures as well as the optical and electronic properties were investigated before and after thermally annealing. All the films show a pure perovskite phase and present typical optical properties of MAPbI_3_. However, after thermal annealing at 145 °C, the decomposed PbI_2_ species occurring at GBs can be revealed in MAPbI_3_ films, leading to a successful passivation at GBs. Therefore, the scattering from GBs, which dominates the charge transport process in the unannealed and 120 °C-annealed MAPbI_3_ films, is obviously suppressed after thermal annealing at 145 °C due to the effective passivation of PbI_2_ that successfully reduces the potential barrier height of GBs. Meanwhile, the scattering from acoustic phonons turns into the prime scattering mechanism in the 145 °C-annealed MAPbI_3_ film. Consequently, Hall mobility is reached to 5 cm^2^/V s, which is significantly higher than that of unannealed one (0.6 cm^2^/V s).

## Data Availability

The datasets used during the current study are available from the corresponding author of this article.

## Abbreviations

GBs: Grain boundaries
HOIPs: Hybrid organic-inorganic perovskites
MAPbI_3_: CH_3_NH_3_PbI_3_
SEM: Scanning electron microscopy
SKPM: Scanning Kelvin probe microscopy
TRPL: Time-resolved photoluminescence
XRD: X-ray diffraction
